# Behavioral Investigation on the Frames of Reference Involved in Visuomotor Transformations during Peripheral Arm Reaching

**DOI:** 10.1371/journal.pone.0051856

**Published:** 2012-12-13

**Authors:** Ettore Ambrosini, Marco Ciavarro, Gina Pelle, Mauro Gianni Perrucci, Gaspare Galati, Patrizia Fattori, Claudio Galletti, Giorgia Committeri

**Affiliations:** 1 Laboratory of Neuropsychology and Cognitive Neuroscience, Department of Neuroscience and Imaging, University “G. d’Annunzio”, Chieti, Italy; 2 Institute of Advanced Biomedical Technologies - ITAB, Foundation G. d’Annunzio, Chieti, Italy; 3 Department of Human and General Physiology and Department of Pharmacy and Biotechnology, University of Bologna, Bologna, Italy; 4 Department of Psychology, Sapienza University of Rome, Rome, Italy; 5 Laboratory of Neuropsychology, Foundation Santa Lucia, Rome, Italy; University of Reading, United Kingdom

## Abstract

**Background:**

Several psychophysical experiments found evidence for the involvement of gaze-centered and/or body-centered coordinates in arm-movement planning and execution. Here we aimed at investigating the frames of reference involved in the visuomotor transformations for reaching towards visual targets in space by taking target eccentricity and performing hand into account.

**Methodology/Principal Findings:**

We examined several performance measures while subjects reached, in complete darkness, memorized targets situated at different locations relative to the gaze and/or to the body, thus distinguishing between an eye-centered and a body-centered frame of reference involved in the computation of the movement vector. The errors seem to be mainly affected by the visual hemifield of the target, independently from its location relative to the body, with an overestimation error in the horizontal reaching dimension (retinal exaggeration effect). The use of several target locations within the perifoveal visual field allowed us to reveal a novel finding, that is, a positive linear correlation between horizontal overestimation errors and target retinal eccentricity. In addition, we found an independent influence of the performing hand on the visuomotor transformation process, with each hand misreaching towards the ipsilateral side.

**Conclusions:**

While supporting the existence of an internal mechanism of target-effector integration in multiple frames of reference, the present data, especially the linear overshoot at small target eccentricities, clearly indicate the primary role of gaze-centered coding of target location in the visuomotor transformation for reaching.

## Introduction

Directing the arm towards a seen object that we want to grasp or touch is a typical example of visuo-motor coordination. Albeit apparently simple, this operation actually requires a series of complex processes. The stimulus position is initially coded by the visual system in retinal coordinates, whereas the motor output guiding the arm movement is coded in intrinsic muscular coordinates. Therefore, the representation of target location must be transformed into coordinates suitable for producing the proper muscle contractions [1]–[3]. Moreover, retinotopic information about target location must be integrated with the position of the effector to compute higher-level movement parameters, such as the direction and distance that the hand must cover to reach the target (movement vector) [4].

To investigate the reference frames involved in arm-movement planning, many psychophysical studies have focused on the spatial pattern of reach errors, basing on the assumption that the error pattern is directly determined by the specific reference frames involved. Several works have found evidence of an oculocentric spatial coding [1], [5]–[8], showing that errors in goal-directed arm-movements vary as a function of the position of the target relative to the current gaze. It has been shown that the spatial position of a reach target is encoded and updated in an eye-centered frame of reference, regardless of whether the target is visual, auditory, tactile or even imaginary [9]. Interestingly, a gaze-centered coding of the location of visual and proprioceptive targets has also been proposed in position judgments [10] and even in tactile localization [11].

These psychophysical results are in accordance with single-unit recordings in monkeys and human functional brain imaging studies, suggesting that a gaze-centered frame of reference is used to represent and update target locations in specific reach-related areas of the posterior parietal cortex (PPC) [12]–[15]. For example, Batista et al. [12] showed that in the parietal reach region (PRR) of the monkey neuronal activity varied when gaze was changed relative to the reach target. More recently, Marzocchi et al. [16] demonstrated that the reach-related activity of area V6A, a reaching area of the medial PPC, was modulated by the retinotopic coordinates of reaching target. Neuropsychological studies on unilateral and bilateral optic ataxia patients (with damage in PPC regions corresponding to monkey PRR and V6A) showed deficits in reaching that are consistent with a dynamic gaze-centered internal representation of reach space. For instance, they have shown that patients with unilateral optic ataxia make large reaching errors when, after foveal target presentation, a saccade prior to movement onset forces them to ‘remap’ the location of the target into their ataxic visual field [17]–[20].

However, other psychophysical experiments have revealed that in the visuomotor transformation process the hand and target positions could be compared also in body-centered coordinates [2], [3], [21]–[25] or in both gaze- and body-centered coordinates [7], [16], [26]–[28]. For instance, in the study of Khan et al. (2007), reaching errors of both control subjects and patients revealed an influence of target position in gaze-centered coordinates, but also a large quasi-independent shoulder-centered influence of target position. Their results thus suggest that, during visuomotor transformations, the target and hand positions are compared in multiple reference frames at more than one level, and these comparisons are then integrated.

The purpose of the present study was to investigate the frames of reference involved in the visuomotor transformation process during reaching movements towards memorized visual targets in space. To this aim, we employed an experimental paradigm that allowed disambiguating the role of eye-centered and body-centered reference frames, by measuring their relative weight in determining subjects’ errors in a reach-to-point task towards the remembered position of visual targets in darkness. This was achieved by experimentally varying the position of the fixation point, as in previous works (e.g., [1]). When only gaze fixation is varied, indeed, the reaching movement remains fixed with respect to the body (both initial hand position and reach target) and errors possibly arising from an intrinsic body-centered representation should remain constant; in contrast, errors arising from a gaze-centered frame of reference should vary depending on gaze direction. Notably, several works have shown that reaching errors vary as a function of the target position relative to current gaze, but it is still unclear if a linear influence does exist (e.g., [1], [29], [30]). To clarify this point, we used several perifoveal target positions. Finally, we also explored the impact of the performing hand on reach errors, an issue which has not been systematically addressed so far.

## Materials and Methods

### Ethics Statement

Participants provided written informed consent before the beginning of the experiment, which was approved by the Ethics Committee of the “G. d’Annunzio” University, Chieti, and was conducted in accordance with the ethical standards of the 1964 Declaration of Helsinki.

### Participants

Twelve human subjects (four males and eight females; mean age±SD = 24.1±1.1 years) participated in the experiment. All participants were right-handed, as defined by the Edinburgh Handedness Inventory [31], without any known neurological or muscular deficits, and had normal or corrected-to-normal vision.

### Apparatus

Subjects were seated on a height-adjustable chair in complete darkness, with the head mechanically stabilized with a chin rest and a head holder, which were mounted onto a wooden table directly in front of them. A Plexiglas screen (120×50 cm) covered with a matte black sheet was placed on the table in a frontal plane within the subject’s reaching distance (at 42 cm). The height of the chair and the chin rest were adjusted so that the subject’s cyclopean eye (located midway between the two eyes) was vertically and horizontally aligned with the central fixation light-emitting diode (LED) (see following section).

**Figure 1:**
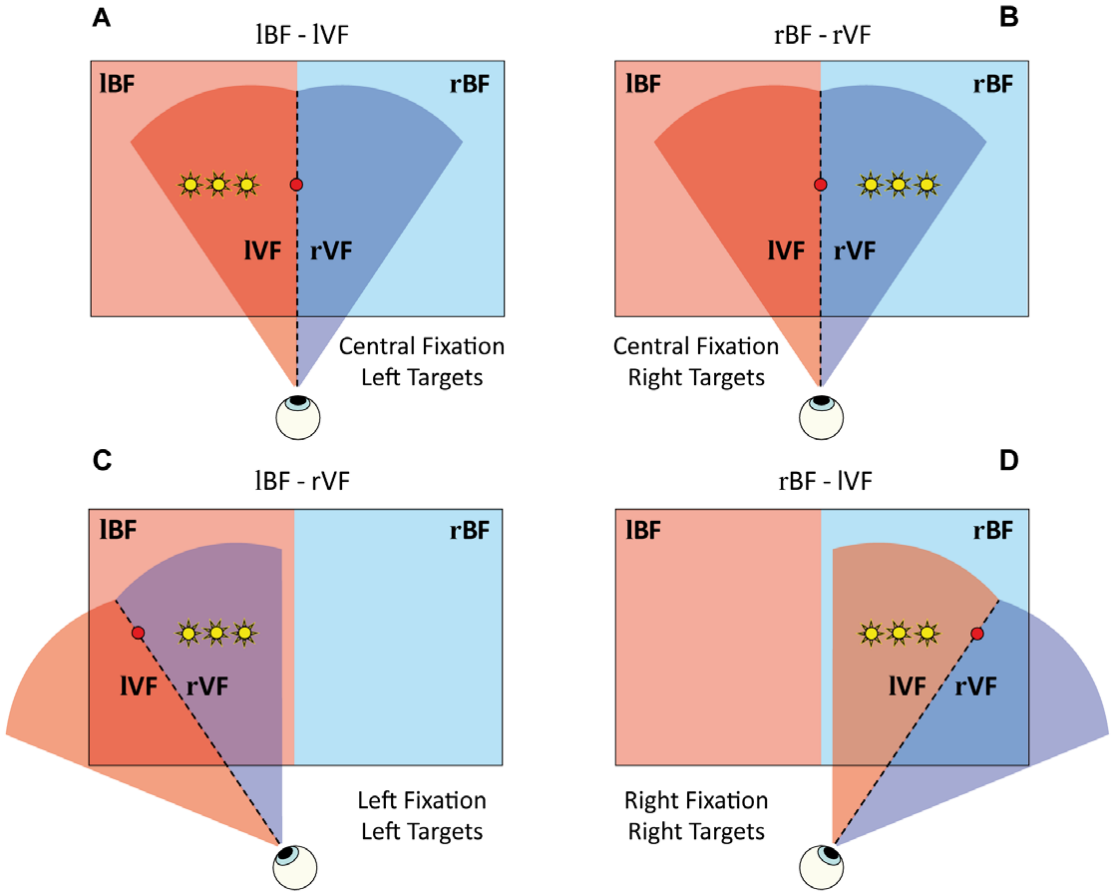
Experimental paradigm. Schematic representation of the experimental paradigm. Red circles represent the three possible red fixation LEDs (left, central, right) while the yellow stars indicate the six target positions used in the entire experiment. Note that three target locations were presented in each of the four conditions. Light red and light blue rectangular areas represent the left and right body fields (BF), respectively, whereas light red and light blue circular sector areas are determined by the fixation point and represent the left and right visual field (VF), respectively. The upper part of the figure illustrates the two experimental conditions with the central fixation, in which the three targets are presented in visual and body compatible fields (panel **A**: left compatibility; panel **B**: right compatibility). The lower part of the figure illustrates the two experimental conditions in which the visual and body hemifields are dissociated by varying the location of the fixation LED. In these cases, the fixation is lateral and the three targets are presented in visual and body incompatible fields (panel **C**: left fixation, left body field but right visual field; panel **D**: right fixation, right body field but left visual field). l = left; r = right.

The stimuli array consisted of nine LEDs aligned on the horizontal plane. Three red LEDs, located at −17.2°, 0°, and 17.2°, served as fixation points. Six yellow LEDs, located at three different eccentricities (11.5°, 8.6°, and 5.7°) on both left and right sides of the central fixation LED, were used as reaching targets (Fig. 1). All LEDs were installed behind the Plexiglas screen. They were visible only when illuminated and gave no tactile feedback when touched. The starting position of the hand reaching movement was a button placed under the chin rest and immediately in front of the subject’s torso.

Movements of the left or the right index finger were monitored using an electromagnetic tracking device (3 Space Fastrak©, Polhemus Navigation; Colchester, VT, USA), which detected the position of small sensors attached to the tip of the left and right index fingers (sampling rate: 120 Hz, static accuracy  = 0.8 mm, resolution  = 0.05 mm). Data were digitized and recorded on a PC for off-line analysis. During the experiment, eye movements were monitored with an infrared tracking system (ISCAN ETL-400, Burlington, MA, USA), which was placed behind the Plexiglas screen.

Stimulus presentation and recording of the participants’ responses were controlled by a custom software (developed by Gaspare Galati at the Department of Psychology, Sapienza Università di Roma, Italy; see [32]), written in MATLAB (The MathWorks Inc., Natick, MA, USA) that implemented Cogent 2000 (developed at FIL and ICN, UCL, London, UK) and Cogent Graphics (developed by John Romaya at the LON, Wellcome Department of Imaging Neuroscience, UCL, London, UK) platforms.

### Experimental Procedures

Participants were requested to reach to the remembered position of a target location in complete darkness, while maintaining fixation at the fixation LED. In order to dissociate the visual from the body spatial coordinates of the reach targets, four experimental conditions were tested by manipulating the position of the fixation LED as illustrated in Figure 1: A) fixation on the central LED and reach targets presented on the left visual field (VF) and left body field (BF) (lVF/lBF: Figure 1A); B) fixation on the central LED and reach targets presented on the right visual and right body fields (rVF/rBF: Figure 1B); C) fixation on the left LED and reach targets presented on the right visual and left body fields (rVF/lBF: Figure 1C); D) fixation on the right LED and reach targets presented on the left visual and right body fields (lVF/rBF: Figure 1D). The four conditions were studied separately in four experimental sessions and, to examine the influence of the performing hand on pointing errors, the four sessions were repeated for both hands. The resulting eight sessions were presented in pseudorandom order for each participant, with the constraint of alternate sessions performed with left and right hand.

At the beginning of each trial, subjects fixated the red fixation LED. Next, one of the yellow reach LEDs (target) was illuminated for 300 ms, while the subject was required to maintain fixation. After a variable delay (200, 300, or 400 ms) from the target offset, the fixation LED flickered, signaling the subjects to reach to and touch the remembered location of the target, while maintaining their gaze fixed at the fixation LED. Reaching movements were performed in darkness and the subjects reported being unable to see their moving arm. Participants were required to complete the reaching movement within 3000 ms, after which the next trial began. For every experimental condition, each of the three reaching targets was presented 16 times in random order, for a total of 48 trials in each session. To prevent darkness adaptation, at the end of every experimental session the room light was switched on for two minutes. Subjects were instructed to perform a fast reaching movement as accurately and fluidly as possible. Before the experiment, subjects completed a brief training session to familiarize with the experimental procedure. The training section lasted until subjects learned to maintain fixation and to move their arm only after the go signal. At the end of the experiment, a calibration procedure was conducted. Participants reached all the LED targets (with visual feedback of the hand) while fixating them. Reaching errors that we report later are computed as the reaching position relative to the corresponding reached position during this calibration procedure.

### Data Analysis

Performance was evaluated by mapping the reaching movement endpoints on the horizontal (x) and vertical (y) axes of the screen. For every trial, endpoint position in the x and y axes was estimated at the point of minimum z position (i.e. the point at which the finger touched the screen). Errors were calculated as the difference between finger endpoint and target position as computed in the calibration procedure.

To quantify movement accuracy we computed three different types of constant errors. The first one, termed “distance” (in cm), was computed as the Euclidean distance between the mean endpoint and target position, and represents the absolute error. The other two measures, named “algebraic x and y errors” (in cm), are equal to the horizontal and vertical component, respectively, of the absolute error and were calculated as the signed difference between the horizontal and vertical components of endpoints and the corresponding values of each target position. “Movement precision” (variable error), instead, was measured by fitting the 95% confidence ellipse on the reaching endpoints distribution separately for each subject for every target and condition. Variable error was then calculated using the area (in cm**^2^**) of these ellipses.

For each dependent measure (mean constant errors and elliptical areas), the statistical significance of the difference between the experimental conditions was tested using repeated-measures analysis of variance (ANOVA) and Newman-Keuls post hoc tests. When the sphericity assumption was violated, we applied Greenhouse-Geisser correction of degrees of freedom (indicated as P**_GG_**).

## Results

The aim of the present study was to investigate the frames of reference used in planning and guiding visuomotor reach-to-touch arm movements. For this purpose, we have examined several measures of accuracy and precision. Each measure was entered as dependent variable in a 2×2×3×2 ANOVA with Visual Field (VF) (lVF vs. rVF), Body Field (BF) (lBF vs. rBF), Target Eccentricity (TE) (5.7°, 8.6°, 11.5°) and performing Hand (lHand vs. rHand) as repeated factors. Data were collected for a total of 4608 trials (384 for each subject). A small percentage of trials (323, i.e. 7% of the total) was discarded off-line because subjects did not maintain fixation or began the arm movement too early (i.e., when movement onset time was less than 100 ms).

### Accuracy (Constant Errors)

**Figure 2:**
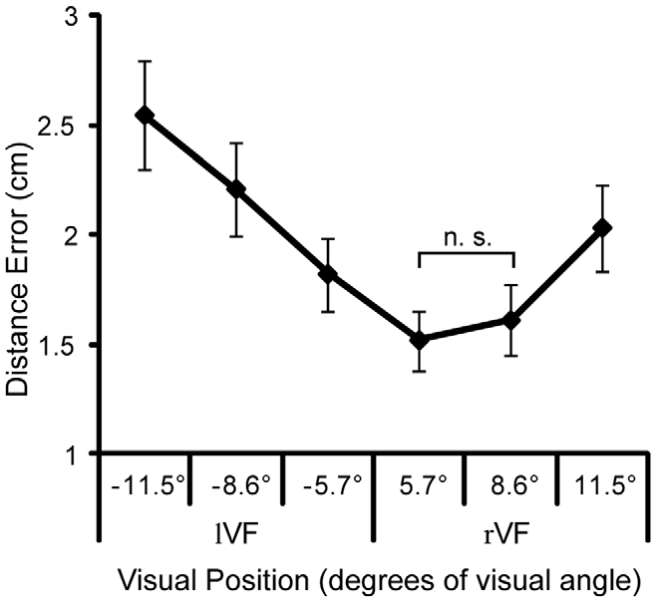
Absolute errors (Distance). 2-way Visual Field × Target Eccentricity interaction. Absolute constant errors are represented as a function of visual position of the targets (i.e., with the eccentricity of the targets located in the left visual field indicated by negative values). Error bars represent standard error of the mean.

The analysis conducted on the absolute constant errors (Distance) showed a clear influence of the oculocentric frame of reference. The ANOVA, indeed, revealed significant main effects of both VF (F_1,11_ = 7.95; P = 0.017), with larger errors in the lVF (M = 2.42 cm, SD = 0.94 cm) than in the rVF (M = 1.90 cm, SD = 0.71 cm), and TE (F_2,22_ = 15.71; P_GG_ = 0.002), with larger errors as target eccentricity increases [M = 1.82, 2.09 and 2.5 cm (SD = 0.58, 0.77 and 1.02 cm) for 5.7°, 8.6° and 11.5° of TE, respectively]. The interaction of these two factors, instead, only approached statistical significance (VF×TE: F**_2,22_** = 3.3; P**_GG_** = 0.056). Post-hoc analysis revealed a stronger influence of target eccentricity in the left visual field (Ps <0.001 for all comparisons), whereas in the right visual field a difference emerged only between targets presented at 11.5° and those presented at 5.7° and 8.6° (Ps <0.001) (Fig. 2).

**Figure 3:**
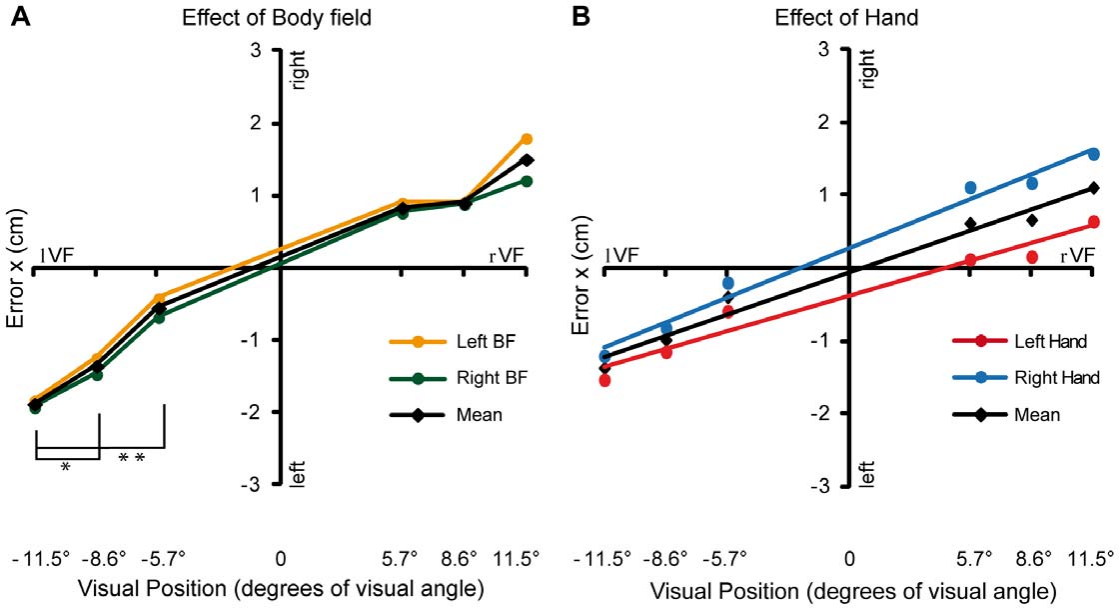
Horizontal errors. (**A**) 2-way Visual Field × Target Eccentricity interaction. The black diamonds represent mean horizontal errors as a function of the visual position of the targets (i.e., with the eccentricity of the targets located in the left visual field indicated by negative values) * indicates P<0.05; ** indicates P<0.01. For illustrative purpose, the data were also split by body field, with the data for the left BF shown in orange, and those for the right body field shown in green. (**B**) Linear regression analyses were computed, for each subject, on the same data of **A** (black diamonds), and on data splitted for performing hand (red and blue circles for left and right hand, respectively). For each of the three regression models, we calculated the mean regression parameters (averaged between subjects); the corresponding three mean regression lines, of the same color of the data points, are superimposed.

The analysis conducted on the horizontal algebraic errors (Fig. 3) revealed a high global accuracy across subjects (x error grand mean = −0.06 cm). The ANOVA revealed the significant main effects of VF (F_1,11_ = 6.04; P = 0.032), TE (F_2,22_ = 7.79; P_GG_ = 0.005) and their interaction (VF×TE: F_2,22_ = 7.05; P_GG_ = 0.021). The main effect of VF showed that the participants systematically overshot the targets (the so-called retinal exaggeration effect; see Discussion section). In other words, subjects made rightward errors when reaching towards the targets located in the right visual field (M = 0.79 cm, SD = 1.02 cm), and leftward errors when reaching towards the left visual field (M = −0.92 cm, SD = 1.75 cm). Post-hoc analysis of the 2-way interaction showed slighter overshooting errors for targets located at lowest eccentricity in the lVF (−5.7° vs. −8.6°: P = 0.044; −5.7° vs. −11.5°: P = 0.005) (Fig. 3A). Note that errors for targets located in the same VF and TE position are not affected by the fact of being in a different BF. On the contrary, within the same BF, the fact of being in a different eye-centered position radically changes the pattern of errors.

Moreover, the ANOVA revealed also a significant main effect of performing Hand (F_1,11_ = 6.69; P = 0.025), with the left hand misreaching towards the left (M = −0.40 cm, SD = 0.92) and the right hand towards the right (M = 0.27 cm, SD = 0.85). This effect seems to be purely additive, since it did not interact with other factors (Fig. 3B).

In order to better clarify the influence of the oculocentric frame of reference on horizontal errors, we applied a linear regression analysis approach evaluating, for each subject, the relationship between the visual position of the targets (in which the three positions with negative values indicate the lVF) and the horizontal error (pooled across hands and BFs). The regression model was significant in most (9 out of 12) of the subjects, predicting that horizontal error is proportional to target position (mean beta coefficient = 0.59; one sample one-tailed t-test against 0: t_11_ = 2.99; P = 0.012). It is also important to note that the intercept (i.e., the error expected for targets presented at the fovea) is not significantly different from zero (t_11_ = −0.29; P>0.77). Moreover, a linear regression was conducted for each hand to verify the additivity of the Hand main effect. Both regression models were significant and explain a large amount of variance in most of the subjects (mean R^2^ = 0.75 and 0.73 for left and right hand, respectively; P<0.05 in 8 out of 12 subjects for both hands). In addition, by comparing the beta coefficients of the two regression models, the regression lines for the two hands were not significantly different (mean beta coefficient = 0.66 and 0.39; two sample two-tailed t-test: t_11_ = −1.86; P = 0.09).

**Figure 4:**
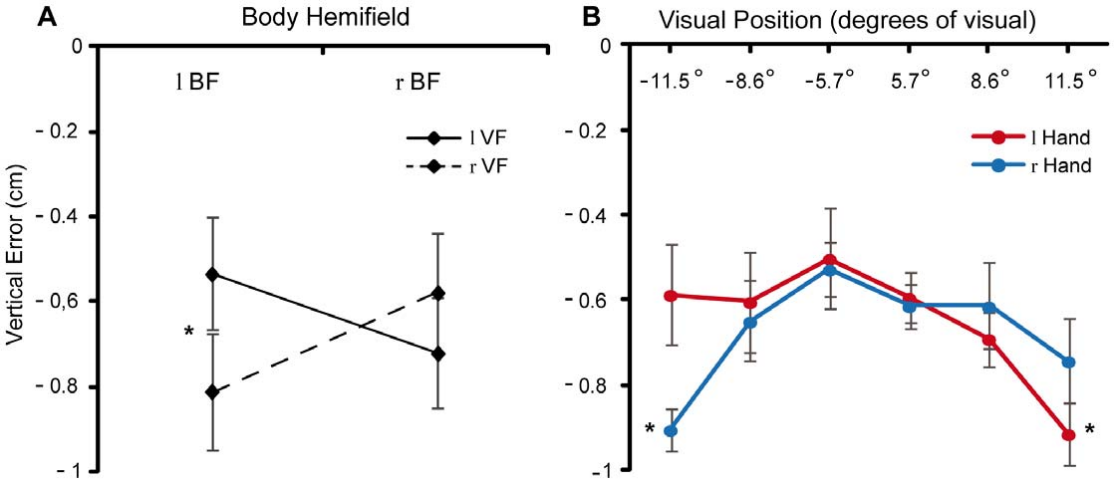
Vertical errors. (**A**) 2-way Visual Field × Body Field interaction (solid line = left visual field; dashed line = right visual field); (**B**) 3-way Hand × Visual Field × Target Eccentricity interaction (red circles = left hand; blue circles = right hand). Error bars represent standard error of the mean.

The analysis conducted on the vertical algebraic errors revealed an overall downward bias (y error grand mean = −0.67 cm) and a significant main effect of target eccentricity (F_2,22_ = 22.03; P_GG_ = 0.0002). Moreover, also the VF×BF 2-way interaction (F_1,11_ = 5.06; P_GG_ = 0.046) and the VF×TE×Hand 3-way interaction (F_2,22_ = 8.07; P_GG_ = 0.005) were significant. Post-hoc analysis of the VF×BF interaction showed that VF affected performance only when targets were presented in the left BF, with subjects making larger errors for targets in the incompatible right VF [rVF = −0.81****cm (SD = 0.77****cm) vs. lVF = −0.53****cm (SD = 0.74****cm); P = 0.05) (Fig. 4A). Post-hoc analysis of the 3-way interaction showed larger errors for targets presented at highest eccentricities, but only when these were located in the visual field opposite to the performing hand [−11.5°: lHand = −0.59****cm (SD = 1.17****cm) Vs. rHand = −0.91****cm (SD = 0.60****cm); 11.5°: lHand = −0.92****cm (SD = 0.70****cm) Vs. rHand = −0.74****cm (SD = 0.98****cm); Ps <0.032) (Fig. 4B). Finally, we investigated the correlation between horizontal and vertical errors, finding that these two types of constant errors were independent (n = 24; r = −0.14; P = 0.5), in line with the pattern of obtained statistical results and with previous findings [1], [5].

### Precision (Variable Errors)

**Figure 5:**
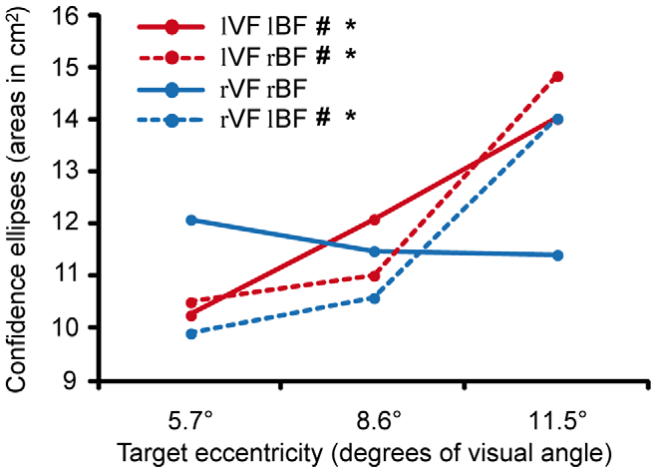
Confidence ellipses areas. 3-way Visual Field × Body Field × Target Eccentricity interaction. The areas of confidence ellipses in the four experimental conditions are represented versus target eccentricity relative to the fixation point (significant post-hoc comparisons are: * = 11.5° vs. 8.6°; # = 11.5° vs. 5.7°). Continuous lines represent compatible visual and body fields, whereas dotted lines represent incompatible visual and body fields (red circles = left hand; blue circles = right hand).

The ANOVA conducted on the finger endpoints distribution area showed the significant main effect of TE (F_2,22_ = 5.71; P_GG_ = 0.01) and the significant VF×BF×TE 3-way interaction (F_2,22_ = 6.20; P_GG_ = 0.007). Post-hoc analysis revealed that ellipse areas for targets located at 11.5° were larger than the other two degrees of Target Eccentricity, except for targets presented in the right compatible condition (rVF/rBF) (Fig. 5).

## Discussion

The main purpose of the present investigation was to shed light on the frames of reference involved in planning and executing a real long-range reaching movement [33] towards visual targets in space. To this aim, we examined the kind of errors performed while subjects reached, in complete darkness and with both hands, memorized targets located at different locations relative to the gaze and/or to the body. This manipulation allowed us to distinguish between an eye-centered and a body-centered frame of reference involved in the computation of the movement vector. At the same time, it allowed us to gain insights into the influence of target eccentricity and performing hand.

### Influence of Visual and Body Fields

The main result indicated that errors were largely influenced by factors associated with the use of an oculocentric frame of reference. We indeed found that two reaching movements show similar errors if target locations are the same in eye-centered coordinates but not if they are identical only in body coordinates.

First, we found that subjects’ accuracy was strongly influenced by the visual hemifield in which targets were presented. More precisely, subjects made horizontal errors that did not depend on target position relative to the screen or their bodies; instead, errors were influenced by target position relative to the fixation point. In other words, when subjects performed a movement towards a target located in the left or right visual hemifield, they made leftward or rightward errors, respectively, regardless of the position of the targets relative to the screen or their bodies. This pattern of errors is well known and mentioned as retinal exaggeration effect [1], [5], [9], [20], [34] or retinal magnification effect [29], and was demonstrated also in more complex tasks in which subjects performed a saccade between the foveal target presentation and the pointing movement towards the stored position of the same targets [1], [8]. These latter works, in particular, suggest that the position of the remembered visual target is not converted into a coordinate system centered on the body or the effector, but is stored and updated in a gaze-centered frame of reference, at least during the preparation of arm movement. It is not clear what exactly produces this systematic mislocalization of targets relative to the gaze. Bock [29] originally described this constant overestimation of reaches relative to the gaze as a magnification of the retinal distance of the target relative to the fovea. Henriques and Crawford [5] suggested that this retinal magnification effect is the result of miscalibration in eye-head coupling when pointing to distant targets with deviated gaze.

Besides confirming the retinal exaggeration effect, the present results indicate a linear correlation between the overestimation errors on the horizontal axis and the target retinal eccentricity (i.e. the distance between target and fixation). Figure 3B shows the good approximation of the linear model to the data, and since the intercept is very close to the origin and not significantly different from zero, no systematic errors were made in reaching the target at the center of the visual field. This suggests that the retinal eccentricity of targets has a linear influence on the representation of targets location in the perifoveal visual field (within 10° from the fovea), an influence that would remain constant upon entering the peripheral visual field (“saturation” effect for targets located beyond 10–15 degrees of eccentricity: [1], [29], [35], [36]). The present new observation of a linear influence within the perifoveal visual field was made possible by the use of several target eccentricities smaller than 10 degrees. Previous studies, which found the saturation effect, employed only one value smaller than 10 degrees, thus preventing such an important observation.

While the effects of the eccentricity discussed so far seem attributable to a systematic bias during the visuomotor transformation process (i.e. impairing subject’s accuracy), the results of the analysis on the dispersion measures suggest that target eccentricity affects also the precision of reaching movements (i.e., increased the variability of subject’s performance). Inspection of Figure 5 shows that increasing the distance between the target and the fixation point results in an increase of endpoint dispersion, regardless of the target distance from the body. In other words, in contrast with previous findings [21], the increase of endpoint dispersion observed for more eccentric targets is not influenced by the distance that the arm has to cover to reach the target.

### Influence of Performing Hand

Besides visual field and target eccentricity effects on accuracy and precision, our work provided further results about an issue which has not been systematically addressed so far: the influence of the performing hand on reaching errors. This influence has been highlighted by the analyses conducted on horizontal errors. These revealed that participants make rightward errors when performing the reaching movement with the right hand, and, conversely, leftward errors with the left hand. In addition, the regression lines calculated for each hand were parallel. These results therefore suggest that the performing hand exerts an influence on the visuomotor transformation processes that is independent from that of the oculocentric frame of reference. The influence of the performing hand on reach errors observed in the present study could be explained by assuming an overestimation bias in proprioceptive localization of the hand starting position [37] that would occur independently of the visuomotor transformation cascade. However, it remains unclear at which stage this influence of the hand can occur. According to the multiple reference model [16], [27], [38]–[40], hand–target information could be compared in multiple reference frames depending on task requirements or available information [41]. Current evidence from neurophysiology, neuroanatomy, and psychophysics strongly supports the existence of multiple, independent, and coexisting levels of representation for combined eye–hand movements in the PPC and connected premotor areas. The parieto-frontal network combines information about target and effector locations during the visuomotor transformation process and neural activity in several parietal and premotor areas appears to be modulated by both hand and target position in different frames of reference [12], [16], [26], [27], [42]–[48]. These results are also consistent with recent findings showing that an artificial neural network of the visuomotor transformation for reaching performs this comparison gradually across different frames of reference [38].

Our data also showed an interesting result that has not been observed in previous works, i.e. a downward bias of reaching errors that was modulated by both target eccentricity and performing hand. Other studies on goal-directed arm-movements showed an overall vertical undershoot of the target position [1], [5], [30], [49], which can be due to a bias toward initial hand position [24] or, more likely, to an interference with the visuomotor transformation by sustained tonus in arm muscles when the arm is raised [49]. Whereas the former hypothesis cannot account for our pattern of errors, since we did not find any bias toward initial hand position in the horizontal component of reaching errors (i.e., an undershoot, instead of an overshoot, in reaching peripheral targets), the latter hypothesis fits better with our results. In fact, a further interference due to a maintained muscle tonus may interact with the imperfect calibration of the retinal read-out, which is the cause of the retinal exaggeration effect, so leading to the target eccentricity modulation of the vertical error that we found.

To conclude, we showed that humans make different errors when reaching to remembered target locations with gaze at different directions. The present results suggest that the location of visual targets is primarily coded in an eye-centered reference frame. Furthermore, our data show that the performance is also influenced by the sensorimotor transformations converting the spatial coordinates of an action target in an independent hand-centered frame of reference. The present results thus support the existence of an internal mechanism of integration between target and effector information in multiple frames of reference. They are in line with the view of a visuomotor transformation in the dorsal visual stream that changes the frame of reference from retinocentric, typically used by the visual system, to arm/hand-centered, typically used by the motor system. It remains a challenge to understand the temporal dynamics of the sensorimotor transformation for reaching implemented by the dorsal visual stream of the human brain.
